# The Effects of Long-Term Chaetomellic Acid A Administration on Renal Function and Oxidative Stress in a Rat Model of Renal Mass Reduction

**DOI:** 10.1155/2017/5125980

**Published:** 2017-02-23

**Authors:** António Nogueira, Francisco Peixoto, Maria Manuel Oliveira, Carlos André Pires, Bruno Colaço, Paula Alexandra Oliveira, Maria João Pires

**Affiliations:** ^1^Department of Veterinary Sciences, School of Agrarian and Veterinary Sciences, University of Trás-os-Montes and Alto Douro (UTAD), Vila Real, Portugal; ^2^Department of Therapeutic and Diagnostic Technologies, Polytechnic Institute of Bragança (IPB), Bragança, Portugal; ^3^Department of Biology and Environment, School of Life Science and Environment, UTAD, Vila Real, Portugal; ^4^Center for the Research and Technology of Agro-Environmental and Biological Science (CITAB), UTAD, Vila Real, Portugal; ^5^Department of Chemistry, School of Life Science and Environment, UTAD, Vila Real, Portugal; ^6^Chemistry Center of Vila Real (CQVR), UTAD, Vila Real, Portugal; ^7^Department of Mathematics, School of Science and Technology, UTAD, Vila Real, Portugal; ^8^Department of Zootechnic, School of Agrarian and Veterinary Sciences, UTAD, Vila Real, Portugal

## Abstract

*Purpose.* This study aimed to evaluate the effect of chronic treatment with chaetomellic acid A (CAA) on oxidative stress and renal function in a model of renal mass reduction.* Methods*. Male Wistar rats were subjected to 5/6 nephrectomy (RMR) or sham-operated (SO). One week after surgery, rats have been divided into four experimental groups: RMR: RMR rats without treatment (*n* = 14); RMR + CAA: RMR rats treated with CAA (*n* = 13); SO: SO rats without treatment (*n* = 13); and SO + CAA: SO rats treated with CAA (*n* = 13). CAA was intraperitoneally administered in a dose of 0.23 *µ*g/Kg three times a week for six months.* Results.* RMR was accompanied by a significant reduction in catalase and glutathione reductase (GR) activity (*p* < 0.05) and a decrease in reduced glutathione (GSH)/oxidized glutathione (GSSG) ratio. CAA administration significantly increased catalase and GR activity (*p* < 0.05) and increased GSH/GSSG ratio, but no significant difference between the treated and nontreated groups was found in this ratio. No significant differences were found between the RMR groups in any of the parameters of renal function. However, CAA administration slightly improves some parameters of renal function.* Conclusions.* These data suggest that CAA could attenuate 5/6 RMR-induced oxidative stress.

## 1. Introduction

Chronic kidney disease (CKD) is a global public health problem associated with increased morbidity, mortality, and health care cost [1]. The major goal for controlling development of CKD is slowing progression to end-stage renal disease. However, slowing its development still represents a clinical challenge [2]. Consequently, the search for new therapeutic modalities remains an issue of actual importance and of interest both to researchers and clinicians.

CKD is characterized by a progressive loss of renal function, chronic inflammation, vascular remodeling, glomerular and tubulointerstitial fibrosis, and oxidative stress [2]. Oxidative stress is implicated in the pathogenesis of renal injury being a constant feature of advanced renal disease and plays a major role in progressive deterioration of renal function and structure [3, 4]. The precise mechanism of CKD-induced oxidative stress has not been completely explained. Impaired oxidative balance in CKD is likely to come from a combination of increased reactive oxygen species (ROS) production and reduced clearance as well as an ineffective antioxidant defense mechanism [2, 5]. The increase of ROS leads to tissue injury and dysfunction by attacking, denaturing, and modifying structural and functional molecules and by activating redox-sensitive transcription factors and the signal transduction pathway. These events, in turn, promote necrosis, apoptosis, inflammation, fibrosis, and other disorders [5].

Farnesyl transferase (FTase) is a zinc enzyme that consists of a 49 kDa *α*-subunit and a 46 kDa *β*-subunit [6]. Initial interest in FTase arose from the discovery that farnesylation is absolutely necessary for the activity of small GTPase protein p21 Ras, Harvey-Ras (Ha-Ras), Neural-Ras (N-Ras), and Kirsten-Ras (Ki-Ras4A and Ki-Ras4B) [7, 8]. Such proteins have a pivotal role in the signal transduction that controls cell growth and differentiation [9] and are involved in the regulation of the intracellular redox state [10–12]. However, different Ras isoforms generate opposing effects on the redox state of cells. Activated Ki-Ras isoform decreases ROS levels by reducing superoxide levels via an activation of the mitochondrial antioxidant enzyme, Mn-superoxide dismutase [12], while activated Ha-Ras isoform increases intracellular levels of ROS via upregulation of the plasma membrane NADPH oxidase that is responsible for the transfer of electrons to molecular oxygen leading to the production of superoxide anions [10, 12–14]. Ha-Ras oxidative stress-induced apoptosis was reversed by farnesyl transferase inhibitors in human umbilical vein endothelial cells [13]. So, one molecular target for prevention oxidative injury associated with CKD may be the Ha-Ras isoform.

Chaetomellic acids are a class of dicarboxylic acids that were isolated from fermentation extract of the coelomycete* Chaetomella acutiseta*. Chaetomellic acid A (CAA) has been identified as potent and highly specific inhibitor of FTase [15], which selectively blocks Ha-Ras farnesylation [16]. CAA has shown to significantly decrease oxidative stress-induced apoptosis in human renal proximal tubular cells and human umbilical vein endothelial cells [17]. Furthermore, CAA administration in rats, after brain damage induced by an excitotoxic stimulus, increased intracellular concentration of nonprenylated inactive Ha-Ras and significantly reduced superoxide production [16]. Additionally, the administration of farnesyl transferase inhibitors, including CAA, reduced renal damage after unilateral ureteral obstruction (UUO) in mice [18]. Ha-Ras proteins were detected in human kidney in different kinds of cells [9]; thus it is reasonable to propose that CAA may protect the kidney from oxidative injury following renal mass reduction.

Therefore, in this study we evaluated the effects of chronic treatment with CAA on renal failure and oxidative stress in rats with 5/6th renal mass reduction.

## 2. Materials and Methods

### 2.1. Animals

Sixty male Wistar rats* (Rattus norvegicus)* (obtained from Harlan Interfauna Iberica, S.L.; Barcelona, Spain) with initial body weights of 359 to 402 g were used in this study. Animals were housed at the University of Trás-os-Montes and Alto Douro facilities under controlled conditions of temperature (23 ± 2°C) and relative humidity (55 ± 5%) on a light-dark cycle (12h : 12h) and provided with a standard laboratory diet (Mucedola, Milan, Italy) and tap water* ad libitum*. All animal procedures were done in accordance with the European Directive 2010/63/EU and the National Decree-Law 113/2013 on the protection of animals used for scientific purposes.

### 2.2. Surgical Procedure and Experimental Groups

After seven weeks of acclimatization, animals were subjected to 5/6th renal mass reduction (RMR) or sham-operated (SO). RMR was performed as described previously [19]. Briefly, the animals were anaesthetized by intraperitoneal injection with ketamine (70 mg/Kg; Imalgene® 1000, Merial S.A.S., Lyon, France) and xylazine (10 mg/Kg; Rompun® 2%, Bayer S.A., Kiel, Germany) and the right kidney was exposed by midline laparotomy, the right renal artery, vein, and ureter were ligated with 4/0 silk suture, and the entire kidney was removed. Then, approximately two-thirds of the left kidney was removed by excision of the upper and lower poles. The weight of the removed renal tissue was measured. After suture of the abdomen, animals were maintained in a warm environment until recovery from anesthesia was complete and then returned to their cages. SO animals underwent a similar procedure. However, only manipulation of the renal pedicles, without any renal mass removal, was performed in these animals. The approximate weight of the remaining renal tissue was calculated on the basis of the removed tissue, assuming that the right and left kidneys had equal weights.

One week after surgical procedure, surviving animals (*n* = 53) were divided into four experimental groups: RMR: RMR rats without treatment (*n* = 14); RMR + CAA: RMR rats treated with CAA (*n* = 13); SO: SO rats without treatment (*n* = 13); and SO + CAA: SO rats treated with CAA (*n* = 13). The rats with renal mass reduction were distributed by the RMR groups according to the serum creatinine concentrations and the weight of the removed renal tissue to ensure equal reduction in renal mass. CAA was intraperitoneally administered in a dose of 0.23 *µ*g/Kg per body weight three times a week for six months. Along the experimental protocol animals were daily observed and animals' body weight was measured weekly during all the experimental protocol. Mortality was also monitored.

### 2.3. Urine and Blood Parameters Measurements

Before and every month after surgery, urine and blood samples were collected. For urine parameters measurements each rat was separately housed in a metabolic cage for two days to get accustomed to isolation conditions, and urine was collected during the following two days to measure volume, urine specific gravity, creatinine concentration, and proteinuria. Blood samples (150 *µ*l) were obtained from a cut in the tail tip into heparinized capillaries. Haematocrit (Ht) was determined in centrifuged capillaries (1500*g* for 15 min) and separated plasma served to measure creatinine, blood urea nitrogen (BUN), phosphorus, and potassium concentrations (Daytona® Rx, Randox) [20]. Glomerular filtration rate was estimated from the creatinine clearance, which was calculated by employing a standard formula [Uc ×* V*/Pc, where Uc = urine creatinine (mg/dl),* V* = urine volume (ml/min/100 g body weight), and Pc = plasma creatinine (mg/dl)].

### 2.4. Blood Pressure Measurement

On the day of sacrifice, the blood pressure was measured. The animals were maintained on surgical anesthesia throughout the entire measurement study with isofluorane. Briefly, the right femoral artery was exposed, and indwelling 24 G catheters (Introcan Safety® G24, B/Braun) were inserted and fixed with 4/0 silk ligatures, taking caution not to twist the vessels. Then, the femoral artery was connected to a Datex-Ohmeda S/5 anesthesia monitor (Datex-Ohmeda Division, Instrumentarium Corp., Helsinki, Finland) through heparin-filled tubes connected to pressure transducers placed at the level of the heart on a plastic support. After being stabilized for 5 min, the values of systolic blood pressure (SBP), diastolic blood pressure (DBP), mean arterial pressure (MAP), and heart rate (HR) were continuously monitored and recorded every 15 sec for a period of 120 sec. At the end of the study the rats were sacrificed using an overdose of anesthesia followed by exsanguination by cardiac puncture as indicated by the Federation of European Laboratory Animal Science Associations [21]. The kidneys were removed and samples were immediately frozen and stored at −80°C until analysis.

### 2.5. Oxidative Stress Studies

#### 2.5.1. Kidney Tissue Homogenate Preparation

The kidneys were weighed and then minced with scissors and homogenized in ice-cold sodium phosphate buffer (KH_2_PO_2_) 100 mM, pH 7.4. The homogenization was carried out in a Potter-Elvejhem homogenizer type after first removing the capsule to obtain 1 : 10 (w/v) dilution. A fraction of the homogenate was stored at −20°C until the evaluation of lipid peroxidation by MDA method and evaluated lipid hydroperoxides. The remaining fraction of the homogenate was centrifuged at 1,500*g* for 10 min at 4°C. The supernatant was then centrifuged at 16,000*g* for 20 min at 4°C and used to determine total protein, catalase (CAT) activity, glutathione reductase (GR) activity, and reduced glutathione (GSH)/oxidized glutathione (GSSG) ratio.

#### 2.5.2. Assessment of Antioxidant Status

The CAT activity was measured with a Clark-type oxygen electrode (Hansatech®) according to Del Río et al. [22]. Assay was conducted as described by Paula Santos et al. [23]. The reaction medium consisted of potassium phosphate buffer (50 mM KH_2_PO_4_ pH 7.4) and H_2_O_2_ (1 M) in a final volume of 1 ml. Medium buffer was previously subjected to a nitrogen stream to decrease the dissolved oxygen. After 2 min of thermostatic incubation at 25°C and stabilization, H_2_O_2_ was added to the reaction medium. Slope was measured and after 30 sec the enzyme extract (diluted 100 times) was added and new slope measured. CAT activity was calculated as mmol H_2_O_2_/min/mg of protein.

The GR activity was performed according to Paula Santos et al. [23] and Smith et al. [24]. The reaction medium consisted of potassium phosphate buffer (100 mM KH_2_PO_4_ and 0.5 mM EDTA, pH 7.4), 100 mM GSSG, and 10 mM NADPH. GSSG was added after 2 min of thermostatic incubation at 25°C to initiate the reaction. GR activity was measured at 340 nm at 25°C by NADPH oxidation. The result was expressed as nmol NADPH oxidized/min/mg of protein.

Concentrations of GSH and GSSG were measured from the supernatant fraction that was obtained in the previously described enzyme assays after ultrasonication and centrifugation. The supernatant, kept at 0°C, was used for the GSH and GSSG assays on the same day. This determination was done by spectrofluorimetry, as previously described [25, 26]. Briefly, GSH was measured by fluorescence using a Varian Eclipse spectrofluorimeter with emission and excitation wavelengths of 426 and 339 nm, respectively, after incubation of 50 *μ*L of supernatant with 1.95 mL of 100 mM K-phosphate buffer (pH 8.0) plus 5 mM EDTA and 200 *μ*L of the fluorescence reagent, o-phthalaldehyde (OPT) (1 mg mL^−1^), for 15 min in the dark and at room temperature (25°C). For GSSG assay, 125 *μ*L of the supernatant was incubated with 50 *μ*L of N-ethylmaleimide 40 mM for 30 min at room temperature (25°C). Thereafter, 140 *μ*L of mixture was incubated with 1.66 mL NaOH 100 mM plus 200 *μ*L OPT solution (1 mg mL^−1^) and incubated for 15 min in the dark and at room temperature (25°C). Fluorescence was determined similarly to that of the GSH assay. Concentrations of GSH and GSSG in the samples were calculated against standard calibration curves.

#### 2.5.3. Assessment of Lipid Peroxidation

Lipid peroxidation was evaluated as thiobarbituric acid reactive products, as previously described by Eriksson et al. [27], and the determination of malondialdehyde (MDA) was based on the derivatization with thiobarbituric acid [28]. The amount of MDA formed was calculated using a molar extinction coefficient of 1.56 × 10^5^ M^−1^ cm^−1^ and expressed as nanomoles MDA per milligram protein.

In order to quantify lipid hydroperoxides, the ferrous oxidation-xylenol orange (FOX) II assay was used; 50 *μ*L of the total lipid extract was added to 950 *μ*L of the FOX reagent solution (100 *μ*M xylenol orange, 250 *μ*M Fe^2+^, 25 mM H_2_SO_4_, and 4 mM 2,6-di-tert-butyl-p-hydroxytoluene (BHT) in 90% (v/v) methanol) in microtubes, homogenized in a vortex mixer, and incubated for 30 min at room temperature, in the dark. After incubation, the absorbance of samples was read at 560 nm against H_2_O_2_ standards with concentrations ranging from 0.0 to 0.4 mM (H_2_O_2_ 1 mM, FOX2 reagent and water). The FOX reagent (100 mL) was prepared as follows: 250 *µ*M (NH_4_)_2_Fe(SO_4_)·6H_2_O (9.8 mg) and 25 mM H_2_SO_4_ (139 *µ*L) were dissolved in 5 mL of water, mixed with 4 mM BHT (88.2 mg), 100 *µ*M xylenol orange (7.2 mg), and 45 mL of methanol. Then, other 45 mL of methanol and 5 mL of water were added [26, 29].

#### 2.5.4. Protein Concentration Measurement

The protein content was determined by the Biuret method with bovine serum albumin as standard [30]. All chemicals used in the enzymatic activity were of analytical purity and were obtained from Sigma Chemical Co. (Sigma Aldrich, San Diego, USA).

### 2.6. Statistical Analysis

Statistical differences between groups were evaluated by one-way analysis of variance (ANOVA) for independent samples, followed by Tukey HSD post hoc tests, when the data was normally distributed. In the other cases the Kruskal-Wallis test was used, followed by multiple comparisons by Dunn's procedure.

For the analysis of the differences over time we used the repeated measures ANOVA, followed by Tukey HSD post hoc tests, when the data was normally distributed, and Friedman's ANOVA for related samples, followed by the Dunn-Bonferroni test for the multiple comparisons, in the other cases. Survival time was estimated using Kaplan-Meier estimates from first day of treatment until death. The four groups were compared using the Log-Rank, if there were an overall difference between the groups; pair-wise comparisons were made among all pairs.

The normality of the data was checked with the Shapiro-Wilk test. All data are presented as mean ± standard error and differences were considered statistically significant for *p* < 0.05.

## 3. Results

### 3.1. Survival

**Figure 1:**
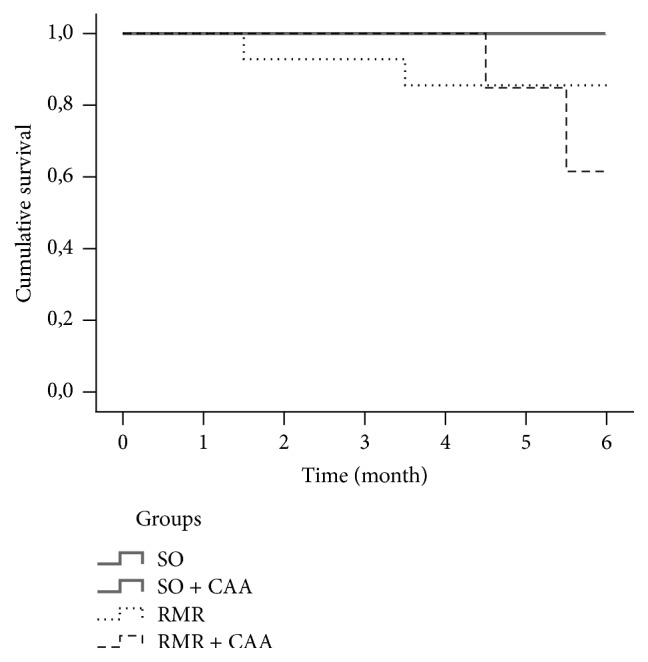
Kaplan-Meier survival analysis. Note significantly reduced survival for group RMR + CAA compared with SO groups (*p* = 0.015).

No mortality was observed in the SO groups throughout the six months of experimental period. Two RMR untreated rats and five RMR animals treated with CAA died during the experimental period (Figure 1). There was a statistical significant difference in cumulative survival between RMR + CAA group and the SO groups.

### 3.2. Blood Pressure and Heart Rate

As expected, SBP, DBP, and MAP were significantly increased in the RMR groups compared with SO groups. Although SBP, DBP, and MAP were lower in the CAA-treated than in the untreated RMR group, the differences were not statistically significant (Table 1). No significant differences were found between the different groups in heart rate (Table 1).

### 3.3. Body Weight and Haematocrit

Table 2 provides the initial and follow-up body weights for all groups. The body weight values of animals belonging to RMR and RMR + CAA groups were similar during the experimental period, and no significant differences were observed between these groups. The haematocrit values of RMR and RMR + CAA groups decreased mainly in the five and six months' time points and were significantly lower than in the SO groups. At sixth month haematocrit value was higher in the RMR + CAA group (46.1 ± 2.0) than in the RMR group (43.5 ± 1.7); however this difference was not statistically significant (Table 2).

### 3.4. Renal Function

The course of the different parameters of renal function in all groups is summarized in Table 2. As we can see, from two-month time point significantly higher plasma levels of BUN and creatinine were observed in the RMR groups compared with the SO groups. Proteinuria was observed as early as one month after renal mass reduction and increased progressively to 3.167 ± 0.410 g/day and 2.467 ± 0.758 g/day urinary protein excretion at six-month time point in the RMR and RMR + CAA groups, respectively, whereas it did not change in the SO groups. Also, from three-month time point creatinine clearance values were significantly lower in the RMR groups compared with the SO groups and reached 0.73 ± 0.10 and 0.92 ± 0.11 ml/min/Kg body weight in the RMR and RMR + CAA groups, respectively, at six-month time point. As shown in Table 2, no significant differences were found between the RMR groups in any of the parameters of renal function. However, at six-month time point the animals belonging to the RMR + CAA group present lower values of urinary flow, phosphorus, potassium, BUN, creatinine, and proteinuria and higher values of urine specific gravity and creatinine clearance than those of the RMR group, but the differences were not statistically significant.

### 3.5. Oxidative Stress

**Figure 2:**
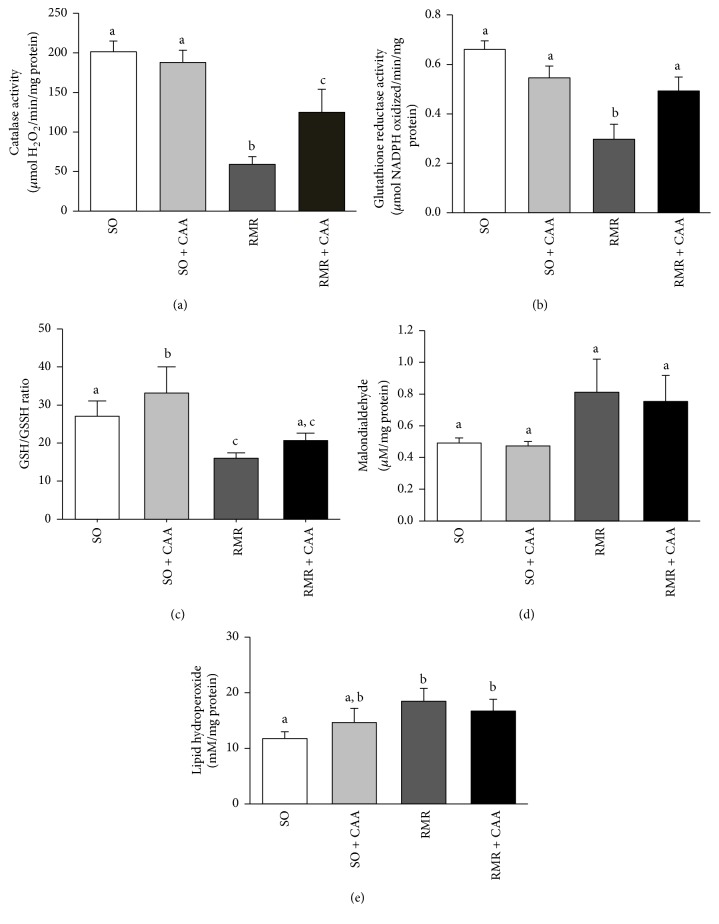
Kidney catalase (a) and glutathione reductase (b) activities, GSH/GSSH ratio (c), malondialdehyde (d), and lipid hydroperoxides (e) levels in experimental groups. ^a,b,c^Differences between groups with the same letter are not statistically significant (*p* < 0.05).

Our next step was to investigate the effects of CAA administration on important cellular antioxidant defenses (Figure 2). It was observed that RMR was accompanied by a reduction in CAT and GR activity and a decrease in GSH/GSSG ratio. CAA administration significantly increased CAT and GR activity and increased GSH/GSSG ratio, but no significant differences between the treated and nontreated groups were found in this ratio. Regarding the oxidative damage to the biological compounds in the renal tissue it is observed in Figure 2 that MDA and lipid hydroperoxides levels, indicators of lipid peroxidation stage, were increased in RMR groups compared with SO groups; however these differences were not statistically significant between RMR and RMR + CAA groups.

## 4. Discussion

CKD is a major health problem worldwide and because oxidative stress plays an important role on its pathogenesis [1, 4], compounds capable of attenuating this process should attract particular interest for evaluation in treating CKD. Therefore, considering that CAA can reduce oxidative stress in human renal proximal tubular cells [17], in the present work, we evaluated different oxidative stress parameters, namely, MDA, lipid hydroperoxides, and GSH/GSSG ratio, as well as the activities of the antioxidant enzymes CAT and GR in homogenates from the kidney of rats subjected to 5/6th nephrectomy, in the hope to contribute to new therapeutic modalities for the treatment of CKD. We also investigated if CAA attenuated the development of renal failure by evaluating urine specific gravity, urinary flow, phosphorus, potassium, BUN, creatinine, proteinuria, and creatinine clearance. To our knowledge, this is the first study evaluating the effect of chronic administration of CAA, a selective inhibitor of membrane-bound Ha-Ras, on renal function and oxidative stress in the kidney of rats with 5/6th renal mass reduction.

Surgical renal mass reduction in rats is a widely studied animal model of chronic renal failure that is close enough to the pathophysiological characteristics of human CKD [31]. This model is characterized by progressive renal failure, proteinuria, and oxidative stress [19, 31–34]. So, we used this model to assess the potential renoprotective effects of CAA.

Experimental data in animal models suggest beneficial effects of antioxidant agents on renal outcomes in CKD [35–40]. In fact, in this study we observed that long-term CAA treatment attenuates oxidative stress in rats with CKD. However, the mortality was higher in animals treated with CAA.

It was observed that although the mortality was higher in the RMR group treated with CAA, the rats died only from the fifth month after RMR. The employed dose of CAA was 0.23 *µ*g/Kg, according to Sabbatini et al. [41]. These authors pretreated the rats with CAA only one time, while we treated our animals with the same dose three times a week for six months. We did not observe adverse effects after the administration of such doses of CAA to rats. Therefore, probably chronic administration of CAA (0.23 *µ*g/Kg) may have some toxicity in rats with enhanced oxidative stress and renal failure, while in healthy animals with a well-balanced redox state and renal function administration of CAA is innocuous. To the best of our knowledge, the toxicological properties of this compound have not been fully investigated. We have measured the renal content of glutathione S-transferase, a phase II detoxification enzyme that plays an important role in elimination of toxic compounds. However, we did not find significant differences between CAA-treated and not treated RMR animals.

Perturbations in cellular oxidant handling influence downstream cellular signaling and, in the kidney, promote renal cell apoptosis and senescence, decreased regenerative ability of cells, and fibrosis. These factors have a stochastic deleterious effect on kidney function [2]. So, typical clinical characteristics of CKD include decreased glomerular filtration rate, proteinuria, and anemia. In this study, progression of renal failure was followed over six months and, as in other studies [19, 42], RMR animals developed renal failure, as assessed by decreased creatinine clearance (an indirect measure of glomerular filtration rate), increased serum creatinine and blood urea nitrogen (BUN), and proteinuria. In general, in animals treated with CAA, all the above alterations were slightly improved, but not statistically significant differences were found between treated and untreated RMR groups.

Oxidative stress in CKD is caused by a combination of excessive ROS production and antioxidant depletion. MDA is an end-product generated by lipid peroxidation and has been used to demonstrate increased oxidative stress during CKD [43, 44]. Several studies demonstrated that renal MDA contents were increased in rats with CKD [32, 42, 45, 46]. Another indicator of lipid peroxidation is the lipid hydroperoxides, well known intermediates of peroxidative reactions [5, 47]. In this study, we found higher oxidative stress levels, as indicated by renal MDA and lipid hydroperoxides concentrations, in the RMR groups compared to the levels observed in SO groups. Although significant differences were not found between RMR groups, MDA and lipid hydroperoxides concentrations were slightly lower in CAA-treated group. In addition, the GSH/GSSG ratio was lower in the RMR group compared with RMR + CAA group. Kim and Vaziri [48] also observed that GSH/GSSG ratio was markedly diminished in rats subjected to 5/6 nephrectomy. Glutathione is a tripeptidic thiol found in the inside of all animal cells and likely is the most important cellular antioxidant. This tripeptide exists predominantly in its reduced form, GSH. GSH is utilized for a broad range of functions, including peroxide clearance and xenobiotic metabolism. Utilization of GSH by antioxidant enzymes generates the oxidized, dimer form of glutathione: GSSG [43]. Thus, determining the ratio of GSH/GSSG is considered a reliable estimate of the degree of cellular oxidative stress. An increase in GSH/GSSG is indicative of augmented antioxidant capacity, whereas a decrease is suggestive of oxidative stress and diminished antioxidant defenses. So, our findings point to increased ROS-induced lipid peroxidation and glutathione oxidation in the RMR groups. However, the degree of these alterations was slightly diminished in animals treated with CAA.

Redox systems including antioxidant enzymes and antioxidant agents provide protection against ROS-induced tissue injury. Glutathione reductase and catalase are among the main enzymatic antioxidants [5, 44]. In this study the administration of CAA was associated to a significantly increased activity of the antioxidant enzymes catalase and glutathione reductase.

Oxidative stress plays an important role in renal lesion induced by 5/6 nephrectomy model [32, 33, 49] and treatment with antioxidants prevents the progression of kidney disease in this model [35, 38–40, 49–52] and preserves the function and structure of the stenotic kidney [53–55]. Significant upregulation of NAD(P)H oxidase, a main source of ROS in the kidney, has been demonstrated in this model [36, 56]. It was established that expression of activated Ha-Ras can increase intracellular levels of ROS via upregulation of the plasma membrane NAD(P)H oxidase [10, 14]. CAA selectively blocks Ha-Ras farnesylation. So, this can explain why the administration of CAA in RMR animals attenuated RMR-induced stress oxidative.

## 5. Conclusions

In the present study, RMR increased the MDA and lipid hydroperoxides levels, decreased renal endogenous antioxidant enzymes, and deteriorated the renal function. Long-term CAA administration (0.23 *µ*g/Kg three times a week, for six months) is associated with a slight reduction of renal MDA and lipid hydroperoxides levels, significant increase in the levels of antioxidant enzymes, and a slight improvement of some parameters of renal function. These data suggest that CAA could attenuate 5/6 RMR-induced oxidative stress mainly by preventing the decrease of antioxidant enzymes. However, potential clinical benefits of CAA therapy require further studies. Although no adverse effect was observed in CAA-treated SO animals, the mortality was higher in RMR animals treated with CAA. Thus, studies on the clinical toxicity and safety of CAA are necessary.

## Figures and Tables

**Table 1:** Blood pressure and heart rate.

Group	SBP	DBP	MAP	HR
SO (*n* = 9)	148.22 ± 6.36	113.00 ± 3.42	128.89 ± 4.24	406.67 ± 5.77
SO + CAA (*n* = 10)	146.00 ± 5.32	111.90 ± 3.15	127.60 ± 3.84	407.00 ± 8.17
RMR (*n* = 9)	202.22 ± 9.61^ab^	140.56 ± 6.28^ab^	167.00 ± 7.54^ab^	420.00 ± 8.33
RMR + CAA (*n* = 7)	184.29 ± 9.79^ab^	133.71 ± 8.48^b^	155.14 ± 8.55^ab^	404.29 ± 13.78

^a^Significant differences compared to SO (*p* < 0.05); ^b^significant differences compared to SO + CAA (*p* < 0.05); SBP: systolic blood pressure; DBP: diastolic blood pressure; MAP: mean arterial pressure; HR: heart rate.

**Table 2:** Body weight, haematocrit, and renal function parameters.

Parameter	Group	Basal	Month 1	Month 2	Month 3	Month 4	Month 5	Month 6
Body weight (g)	SO (*n* = 13)	392 ± 10	458 ± 12	484 ± 13^0^	506 ± 13^01^	539 ± 15^012^	551 ± 16^0123^	561 ± 16^01234^
	SO + CAA (*n* = 13)	371 ± 5	435 ± 5	462 ± 6^0^	487 ± 7^01^	524 ± 9^012^	534 ± 10^0123^	550 ± 10^0123^
	RMR (*n* = 12)	370 ± 11	412 ± 10^a^	444 ± 13^0^	478 ± 14^01^	513 ± 14^0123^	517 ± 14^0123^	516 ± 13^0123^
	RMR + CAA (*n* = 8)	374 ± 7	407 ± 10^ab^	446 ± 10	463 ± 11^0^	491 ± 10^012^	494 ± 12^a012^	503 ± 12^a0123^

Haematocrit (%)	SO (*n* = 13)	49.9 ± 0.5	55.4 ± 0.6	54.3 ± 0.6^0^	53.0 ± 0.7^1^	54.9 ± 0.5^0^	54.7 ± 0.5^0^	54.8 ± 0.7^0^
	SO + CAA (*n* = 13)	48.2 ± 0.6	54.9 ± 0.8^0^	54.0 ± 0.7^0^	55.1 ± 0.7^0^	53.4 ± 0.7^0^	49.8 ± 1.6^a123^	53.0 ± 0.7^0^
	RMR (*n* = 12)	49.0 ± 0.5	49.3 ± 0.5^ab^	49.4 ± 0.7^ab^	49.2 ± 0.8^ab^	48.9 ± 0.9^ab^	46.2 ± 0.9^ab01234^	43.5 ± 1.7^ab01234^
	RMR + CAA (*n* = 8)	48.2 ± 0.6	51.8 ± 0.5^ab^	52.5 ± 0.7	51.4 ± 0.8^b^	51.3 ± 1.2^a^	45.9 ± 1.2^ab1234^	46.1 ± 2.0^ab1234^

Urine specific gravity	SO (*n* = 13)	1.035 ± 0.002	1.035 ± 0.002	1.032 ± 0.002	1.035 ± 0.002	1.037 ± 0.001	1.039 ± 0.001^2^	1.039 ± 0.001^2^
	SO + CAA (*n* = 13)	1.033 ± 0.002	1.038 ± 0.0010	1.035 ± 0.001^1^	1.037 ± 0.001	1.038 ± 0.001^2^	1.041 ± 0.000^023^	1.040 ± 0.000^23^
	RMR (*n* = 12)	1.029 ± 0.002	1.027 ± 0.002^ab^	1.024 ± 0.002^ab^	1.025 ± 0.002^ab^	1.025 ± 0.001^ab^	1.027 ± 0.001^ab^	1.026 ± 0.001^ab^
	RMR + CAA (*n* = 8)	1.029 ± 0.004	1.032 ± 0.002^b^	1.030 ± 0.002	1.028 ± 0.002^ab^	1.028 ± 0.002^ab^	1.031 ± 0.002^ab^	1.030 ± 0.002^ab^

Urinary flow (ml/hour)	SO (*n* = 13)	0.60 ± 0.06	0.61 ± 0.06	0.74 ± 0.07^01^	0.60 ± 0.06^2^	0.67 ± 0.05	0.69 ± 0.04^013^	0.64 ± 0.04
	SO + CAA (*n* = 13)	0.57 ± 0.04	0.53 ± 0.05	0.54 ± 0.04	0.50 ± 0.05	0.72 ± 0.05^013^	0.60 ± 0.03^34^	0.56 ± 0.05^4^
	RMR (*n* = 12)	0.78 ± 0.05	1.01 ± 0.06^ab^	1.11 ± 0.09^ab^	1.09 ± 0.08^ab^	1.51 ± 0.12^ab0123^	1.66 ± 0.12^ab0123^	1.76 ± 0.14^ab0123^
	RMR + CAA (*n* = 8)	0.76 ± 0.20	0.80 ± 0.07^b^	0.88 ± 0.09^b^	0.92 ± 0.09^ab^	1.10 ± 0.10^ab01^	1.43 ± 0.18^ab0123^	1.49 ± 0.20^ab0123^

Phosphorus (mg/dl)	SO (*n* = 13)	5.97 ± 0.10	6.31 ± 0.15	6.01 ± 0.13	5.56 ± 0.20^1^	6.02 ± 0.20	5.68 ± 0.10^1^	6.39 ± 0.28^35^
	SO + CAA (*n* = 13)	6.13 ± 0.15	6.55 ± 0.24	5.85 ± 0.20^1^	6.01 ± 0.18^1^	6.11 ± 0.17	5.49 ± 0.17^014^	6.07 ± 0.28^5^
	RMR (*n* = 12)	5.69 ± 0.12	6.22 ± 0.21^1^	5.09 ± 0.16^ab1^	5.48 ± 0.20^1^	5.97 ± 0.14^2^	6.05 ± 0.11^2^	8.21 ± 0.49^ab012345^
	RMR + CAA (*n* = 8)	6.08 ± 0.13	6.78 ± 0.15	5.87 ± 0.12^c1^	5.94 ± 0.19^1^	5.75 ± 0.34^1^	5.89 ± 0.66^1^	7.48 ± 0.48^b02345^

Potassium (mmol/l)	SO (*n* = 13)	5.44 ± 0.13	4.82 ± 0.09^0^	5.07 ± 0.10	4.69 ± 0.10^0^	5.55 ± 0.13^123^	5.34 ± 0.11^13^	4.81 ± 0.10^045^
	SO + CAA (*n* = 13)	5.33 ± 0.08	5.13 ± 0.13	5.24 ± 0.12	5.03 ± 0.10	5.08 ± 0.14	5.20 ± 0.09	4.51 ± 0.18^01245^
	RMR (*n* = 12)	5.17 ± 0.07	5.32 ± 0.05^a^	5.00 ± 0.13	5.05 ± 0.11	5.16 ± 0.09	5.28 ± 0.12	5.28 ± 0.14^b^
	RMR + CAA (*n* = 8)	5.40 ± 0.05	5.65 ± 0.13^ab^	5.38 ± 0.18	4.92 ± 0.16^1^	4.85 ± 0.21^a01^	5.27 ± 0.16	4.85 ± 0.14^01^

BUN (mg/dl)	SO (*n* = 13)	28.8 ± 1.1	56.5 ± 2.3^0^	30.6 ± 1.8^1^	34.3 ± 0.7^1^	37.8 ± 0.8^012^	42.9 ± 1.3^023^	40.4 ± 1.9^012^
	SO + CAA (*n* = 13)	28.4 ± 1.1	35.6 ± 1.4^a^	34.0 ± 1.3	34.8 ± 1.7	34.3 ± 1.0	50.2 ± 2.1^01234^	45.7 ± 2.0^01234^
	RMR (*n* = 12)	26.1 ± 1.1	70.8 ± 2.9^ab0^	91.2 ± 5.1^ab01^	63.9 ± 4.0^ab02^	66.5 ± 3.5^ab02^	90.9 ± 6.7^ab034^	146.7 ± 20.2^ab0134^
	RMR + CAA (*n* = 8)	28.0 ± 1.8	66.2 ± 2.0^b0^	96.3 ± 3.6^ab01^	56.4 ± 4.8^ab02^	63.9 ± 4.0^ab02^	76.0 ± 6.0^ab0^	95.9 ± 14.2^ab034^

Plasma creatinine (mg/dl)	SO (*n* = 13)	0.68 ± 0.02	0.54 ± 0.01^0^	0.61 ± 0.01^0^	0.70 ± 0.02^12^	0.74 ± 0.02^12^	0.74 ± 0.02^12^	0.74 ± 0.02^12^
	SO + CAA (*n* = 13)	0.69 ± 0.02	0.90 ± 0.05^a0^	0.60 ± 0.02^1^	0.64 ± 0.02^1^	0.67 ± 0.02^1^	0.72 ± 0.01^12^	0.75 ± 0.05^12^
	RMR (*n* = 12)	0.68 ± 0.02	0.98 ± 0.04^a0^	0.78 ± 0.04^ab1^	0.92 ± 0.06^ab0^	0.92 ± 0.03^ab0^	1.06 ± 0.07^ab02^	1.69 ± 0.23^ab012345^
	RMR + CAA (*n* = 8)	0.74 ± 0.02	1.01 ± 0.03^a0^	0.85 ± 0.03^ab1^	0.91 ± 0.04^ab0^	0.95 ± 0.03^ab0^	1.00 ± 0.05^ab01^	1.28 ± 0.18^ab013^

Proteinuria (g/day)	SO (*n* = 13)	0.098 ± 0.005	0.108 ± 0.007	0.108 ± 0.005	0.093 ± 0.008	0.100 ± 0.004	0.106 ± 0.006	0.105 ± 0.006
	SO + CAA (*n* = 13)	0.095 ± 0.009	0.130 ± 0.012^0^	0.110 ± 0.008	0.096 ± 0.008^12^	0.112 ± 0.007	0.107 ± 0.005^1^	0.117 ± 0.008^035^
	RMR (*n* = 12)	0.110 ± 0.012	0.194 ± 0.014^ab^	0.326 ± 0.063^ab0^	0.578 ± 0.159^ab0^	1.117 ± 0.286^ab012^	1.997 ± 0.363^ab01234^	3.167 ± 0.410^ab012345^
	RMR + CAA (*n* = 8)	0.088 ± 0.010	0.179 ± 0.017^ab^	0.290 ± 0.047^ab0^	0.433 ± 0.132^ab0^	0.688 ± 0.210^ab0^	1.360 ± 0.462^ab01234^	2.467 ± 0.758^ab012345^

Creatinine clearance (ml/min)	SO (*n* = 13)	1.67 ± 0.17	1.55 ± 0.07	1.62 ± 0.09	1.57 ± 0.09	1.68 ± 0.07	1.84 ± 0.08	1.56 ± 0.04
	SO + CAA (*n* = 13)	1.55 ± 0.09	0.90 ± 0.06^a0^	1.42 ± 0.06^1^	1.44 ± 0.061	1.71 ± 0.03^123^	1.60 ± 0.05^1^	1.45 ± 0.09^14^
	RMR (*n* = 12)	1.70 ± 0.11	0.77 ± 0.04^a0^	1.09 ± 0.07^ab01^	1.10 ± 0.07^ab01^	1.14 ± 0.06^ab01^	1.27 ± 0.07^ab01^	0.73 ± 0.10^ab02345^
	RMR + CAA (*n* = 8)	1.63 ± 0.41	0.79 ± 0.04^a0^	0.97 ± 0.05^ab0^	1.09 ± 0.05^ab01^	1.13 ± 0.07^ab01^	1.28 ± 0.04^ab12^	0.92 ± 0.11^ab05^

^a^Significant differences compared to SO;  ^b^significant differences compared to SO + CAA;  ^c^significant differences compared to RMR.

^0^Significant differences compared to basal (*p* < 0.05);  ^1^significant differences compared to first month (Month 1) (*p* < 0.05);  ^2^significant differences compared to second month (Month 2) (*p* < 0.05);  ^3^significant differences compared to third month (Month 3) (*p* < 0.05);  ^4^significant differences compared to fourth month (Month 4) (*p* < 0.05);  ^5^significant differences compared to fifth month (Month 5) (*p* < 0.05).
